# An Improved Canonical Correlation Analysis for EEG Inter-Band Correlation Extraction

**DOI:** 10.3390/bioengineering10101200

**Published:** 2023-10-16

**Authors:** Zishan Wang, Ruqiang Huang, Ye Yan, Zhiguo Luo, Shaokai Zhao, Bei Wang, Jing Jin, Liang Xie, Erwei Yin

**Affiliations:** 1 School of Information Science and Engineering, East China University of Science and Technology, Shanghai 200030, China; w2972083043@163.com (Z.W.); beiwang@ecust.edu.cn (B.W.); jinjing@ecust.edu.cn (J.J.); 2 Defense Innovation Institute, Academy of Military Sciences (AMS), Beijing 100000, China; zhiguo_luo_nudt@foxmail.com (Z.L.); lnkzsk@126.com (S.Z.); xielnudt@gmail.com (L.X.); yinerwei1985@gmail.com (E.Y.); 3 Tianjin Artificial Intelligence Innovation Center (TAIIC), Tianjin 300450, China; huang_rq17@126.com

**Keywords:** EEG, IBC, CCA, DE, decision-level fusion

## Abstract

(1) Background: Emotion recognition based on EEG signals is a rapidly growing and promising research field in affective computing. However, traditional methods have focused on single-channel features that reflect time-domain or frequency-domain information of the EEG, as well as bi-channel features that reveal channel-wise relationships across brain regions. Despite these efforts, the mechanism of mutual interactions between EEG rhythms under different emotional expressions remains largely unexplored. Currently, the primary form of information interaction between EEG rhythms is phase–amplitude coupling (PAC), which results in computational complexity and high computational cost. (2) Methods: To address this issue, we proposed a method of extracting inter-bands correlation (IBC) features via canonical correlation analysis (CCA) based on differential entropy (DE) features. This approach eliminates the need for surrogate testing and reduces computational complexity. (3) Results: Our experiments verified the effectiveness of IBC features through several tests, demonstrating that the more correlated features between EEG frequency bands contribute more to emotion classification accuracy. We then fused IBC features and traditional DE features at the decision level, which significantly improved the accuracy of emotion recognition on the SEED dataset and the local CUMULATE dataset compared to using a single feature alone. (4) Conclusions: These findings suggest that IBC features are a promising approach to promoting emotion recognition accuracy. By exploring the mutual interactions between EEG rhythms under different emotional expressions, our method can provide valuable insights into the underlying mechanisms of emotion processing and improve the performance of emotion recognition systems.

## 1. Introduction

Emotion is an essential medium of communication between people and the outside world, reflecting human physiological and psychological states, and influencing behavior and decision-making directly. In recent years, with the rapid development of human–computer interaction (HCI), affective computing has emerged as a research hotspot. Affective computing aims to empower computers with the ability to perceive, understand, and express human emotions, enhancing their intelligence. As the core of affective computing [1], emotion recognition has become increasingly critical. It holds significant importance in diagnosing emotion-related diseases and constructing harmonious and friendly human–computer interaction systems.

Current emotion recognition methods can be mainly divided into two categories. One is based on non-physiological signals, such as facial expressions [2,3], body movements [4] and speech signals [5,6]. The other type is based on physiological signals, such as Electroencephalography (EEG) [7,8], Electrocardiogram (ECG) [9,10], Electromyography (EMG) [11] and galvanic skin response (GSR) [12]. Compared with non-physiological signals, physiological signals have the advantage of being difficult to disguise, so they can reflect people’s emotional states more objectively. Among the numerous physiological signals, EEG is the signal most frequently used for emotion recognition because it can dynamically reflect changes in the central nervous system and contains rich emotional information [13].

In the emotion recognition task, the quality of feature extraction [14] affects its performance directly. For EEG signals, the features commonly used for emotion recognition mainly include time domain [15,16,17], frequency domain [15] and time–frequency domain [15,18] features. Duan proposed a feature called differential entropy (DE) [19]. The experimental results on subjects show that differential entropy is an effective feature for identifying emotions and outperforms traditional power spectral density (PSD) [20] features. Kamble [21] developed an integrated eigenvector centrality-variational nonlinear chirp mode decomposition-based EEG rhythm separation (EVNCERS) and analyzed the rhythms associated with several entropy-based features, which were designed to accurately detect affective emotions from various frequency bands of EEG rhythms. Vanitha [22] used the Hilbert–Huang transform (HHT) to detect EEG-related emotional patterns in the time–frequency domain and classified emotions using a multi-class support vector machine classifier (MC-SVM). The results demonstrate the effectiveness of the proposed time–frequency analysis method when it comes to detecting emotions using EEG signals. In addition, some studies suggest that the relationship between EEG channels is also a feature that needs to be considered. Moon [23] identified emotions by extracting the connectivity features between different brain channels, including Pearson correlation coefficient (PCC), phase lock value (PLV) and phase lag index (PLI). They constructed a connectivity matrix as the input of the convolutional neural network (CNN) by calculating the connectivity coefficient between any two EEG channels. The results showed that connectivity between the EEG channels was indeed an important feature of emotion, reflecting the synergy between different brain regions as emotions arise and change. Further, some senior features were extracted. Khare proposed a novel time-order representation (TOR) based on the S-transform for the identification of human emotions. This TOR was given as an input to CNN to automatically extract and classify the deep features [24], which proved that deep features extracted using this method were effective for emotion recognition.

However, the mechanism of mutual interactions between EEG frequency bands under different emotional expressions lacks full exploration. Theoretically, the generation of emotion is accompanied by the common activity of multiple frequency bands. Currently, the main way of information interaction between EEG frequency bands is phase–amplitude coupling (PAC) and it is indicated that PAC is a potential feature for emotion [25]. However, the high computational complexity of PAC is a huge challenge to the real-time performance of the model. Aru [26] indicated that a critical step of the typical approach to analyzing PAC was statistical evaluation. Because there is no direct way to quantify the significance of PAC scores, one needs to rely on statistical inference to reach a conclusion about the statistical significance of the measure. Presently, most studies of PAC rely on the frequentist approach of using surrogate data to estimate a *p*-value. Hülsemann [27] illustrated the method of surrogate testing. During surrogate testing, the observed coupling value is compared to a distribution of shuffled coupling values. Shuffling is usually repeated 200 to 1000 times, which leads to high computational complexity and is time consuming. In summary, the current research gap is that there is a lack of sufficient research on the cross-frequency coupling, and there is great room for improvement in the extraction of relevant emotional features between frequency bands, whether in terms of model accuracy or time cost.

Therefore, in this paper, we consider the synergy between EEG frequency bands. First, we extracted the DE features of each frequency band of the multi-channel EEG signals. Then, we extracted the correlating features between the EEG frequency band through canonical correlation analysis (CCA) [28,29] based on DE. Finally, we used the DE feature and the correlation feature between frequency bands to train a support vector machine (SVM) classifier, respectively, and integrated the two classifiers to identify emotions through decision-level fusion, which significantly improves the accuracy of emotion recognition compared with that of a single feature.

The other parts of this paper are arranged as follows: In Section 2, we describe in detail the dataset we used and the preprocessing, feature extraction, decision fusion, and cross-validation methods employed when analyzing the data. In Section 3, the experimental results are presented and some analysis and discussion are carried out. Section 4 summarizes the main conclusions and contributions of our study.

## 2. Materials and Methods

### 2.1. Datasets and Preprocessing

We used two datasets: the SJTU Emotion EEG Dataset (SEED) [30] and our local Continuous Upgrading Multi-Model Affect Elicitation (CUMULATE) dataset [31].

The SEED dataset consists of 15 participants (7 males and 8 females, mean age 23.27 years, variance 2.37). Each subject participated in three experiments, and in each experiment, they were required to watch 15 emotional film clips to elicit three emotions: positive, neutral, and negative. This dataset recorded EEG signals from 62 electrodes (FPZ, FP1, FP2, AF3, AF4, F7, F5, F3, F1, FZ, F2, F4, F6, F8, FT7, FC5, FC3, FC1, FCZ, FC2, FC4, FC6, FT8, T7, C5, C3, C1, CZ, C2, C4, C6, T8, TP7, CP5, CP3, CP1, CPZ, CP2, CP4, CP6, TP8, P7, P5, P3, P1, PZ, P2, P4, P6, P8, PO7, PO5, PO3, POZ, PO4, PO6, PO8, CB1, O1, OZ, O2, CB2) with ESI NeuroScan System. The acquired EEG signals were then preprocessed. In order to filter out noise and artifacts, the signals were processed using a bandpass filter from 0.3 to 50 Hz, followed by downsampling of the sampling rate from 1000 Hz to 200 Hz. Afterwards the EEG signal corresponding to the video clip was segmented and the data of each channel were divided into 5 s lengths without overlap.

The CUMULATE dataset is our local dataset, containing a three-classes sub-dataset of calm, fear and happy; a four-classes sub-dataset of calm, fear, happy and sad; and a five-classes sub-dataset of calm, fear, happy, sad and angry. Each sub-dataset contained 15 subjects, 5 of whom participated in both rounds of the experiment, so a total of 40 subjects participated in the experiment (30 males and 10 females, mean age 26.20 years, variance 4.74). In each type of sub-dataset, there were five video clips for each emotion. Thus 15, 20, and 25 video clips were viewed by subjects in the three-classes, four-classes, and five-classes sub-dataset, respectively. A total of 60 EEG channels (FPZ, FP1, FP2, AF3, AF4, F7, F5, F3, F1, FZ, F2, F4, F6, F8, FT7, FC5, FC3, FC1, FCZ, FC2, FC4, FC6, FT8, T7, C5, C3, C1, CZ, C2, C4, C6, T8, TP7, CP5, CP3, CP1, CPZ, CP2, CP4, CP6, TP8, P7, P5, P3, P1, PZ, P2, P4, P6, P8, PO7, PO5, PO3, POZ, PO4, PO6, PO8, O1, OZ, O2) were recorded with the ESI NeuroScan System. After that, the same bandpass filtering process of 0.3–50 Hz was performed on the original EEG signal to remove the high-frequency noise, and a notch filter of 50 Hz was used to remove power line interference. The signal was then downsampled to 200 Hz for subsequent processing. To avoid the interference of eye movement artifacts, the eye movement artifacts were removed via independent component analysis (ICA) [32]. Similarly, the pre-processed EEG signal was segmented. The data of each channel were divided into 5 s lengths without overlap.

### 2.2. Differential Entropy

For EEG signals, DE is a very effective feature, which is a generalized form of Shannon information entropy over continuous variables [19,30]. Assuming that X is a continuous random variable, the differential entropy is calculated using the formula shown in Equation (1):

(1) $$DE=-\int_{X}^{}p\left(x\right)\log\left(p\left(x\right)\right)dx$$

where $p\;\left(x\right)$ is the probability density function of the random variable X. When the random variable $X\sim N\;(\mu ,\sigma^{2})$, Equation (1) can be rewritten as Equation (2):

(2) $$DE=-\int_{-\infty }^{\infty }\frac{1}{\sqrt{2\pi \sigma^{2}}}e^{-\frac{{\left(x-\mu \right)}^{2}}{2\sigma^{2}}}log\left(\frac{1}{\sqrt{2\pi \sigma^{2}}}e^{-\frac{{\left(x-\mu \right)}^{2}}{2\sigma^{2}}}\right)dx=\frac{1}{2}log\left(2\pi e\sigma^{2}\right)$$

Studies have shown that for a fixed-length EEG signal, the differential entropy of the EEG signal in a frequency band is equivalent to the logarithm of the energy spectrum in the frequency band [33]. Thus, we can calculate the differential entropy of five frequency bands (δ: 1–4 Hz, θ: 4–8 Hz, α: 8–13 Hz, β: 13–30 Hz, γ: 30–50 Hz) per channel for each sample. For the SEED dataset, we can extract 310 dimensional features (62 channels × 5 bands) for each sample, and for the CUMULATE dataset, we can extract 300 dimensional features (60 channels × 5 bands) for each sample.

### 2.3. Canonical Correlation Analysis

CCA is usually used to study the correlation between two sets of multidimensional random variables. It is an extension of the correlation between two random variables under two sets of random variables [34]. For two random variables x and y, the correlation coefficients are defined as follows:

(3) $$\rho =\frac{Cov\left(x,y\right)}{\sqrt{D\left(x\right)D\left(y\right)}}$$

where $Cov\left(x,y\right)$ is the covariance of x, y and $\sqrt{D\left(x\right)D\left(y\right)}$ is the variance of x, y. For two sets of random variables X and Y, CCA discusses the correlation by finding a certain linear combination of the two sets of variables, respectively. As shown in Equations (4) and (5):

(4) $$x^{\prime }=a^{T}X$$

(5) $$y^{\prime }=b^{T}Y$$

where $x^{\prime }$ and $y^{\prime }$ is the linear combination of X and Y. The goal of CCA is to find the values of a and b that maximize the correlation coefficient between $x^{\prime }$ and $y^{\prime }$. As follows:

(6) $$\underset{a,b}{\mathrm{argmax}}\rho =\underset{a,b}{\mathrm{argmax}}\frac{Cov\left(a^{T}X,b^{T}Y\right)}{\sqrt{D\left(a^{T}X\right)D\left(b^{T}Y\right)}}$$

Because X and Y are preprocessed multi-dimensional variables with a mean of 0 and a variance of 1, Equation (6) can be transformed into Equation (7):

(7) $$\underset{a,b}{\mathrm{argmax}}\frac{a^{T}Cov\left(X,Y\right)b}{\sqrt{a^{T}D\left(X\right)a}\sqrt{b^{T}D\left(Y\right)b}}$$

Since the numerator and denominator are increased by the same multiple, the optimization objective result remains unchanged, so our optimization objective can be transformed into the following:

(8) argmaxa,b⁡aTCovX,Ybs.t.  aTDXa=bTDYb=1

For the optimization objective of the above formula, it can be solved via SVD decomposition. Let $S_{XY}=Cov\left(X,Y\right)$, $S_{XX}=D\left(X\right),\;{\;S}_{YY}=D\left(Y\right)$ to simplify the writing. Firstly, Let $a=S_{XX}^{-1/2}u$, $b=S_{YY}^{-1/2}v$. So:

(9) $$a^{T}S_{XX}a=u^{T}S_{XX}^{-1/2}S_{XX}S_{XX}^{-1/2}u=u^{T}u=1$$

(10) $$b^{T}S_{YY}b=v^{T}S_{YY}^{-1/2}S_{YY}S_{YY}^{-1/2}v=v^{T}v=1$$

(11) $$a^{T}S_{XY}b=u^{T}S_{XX}^{-1/2}S_{XY}S_{YY}^{-1/2}v$$

Therefore, our optimization objective transform into Equation (12):

(12) argmaxa,b⁡uTSXX−1/2SXYSYY−1/2vs.t.  uTu=vTv=1

Let $M=S_{XX}^{-1/2}S_{XY}S_{YY}^{-1/2}$, and treat u and v as left and right singular vectors corresponding to a singular value of matrix M. Performing singular value decomposition on M gives us $M=U\Sigma V^{T}$, where U and V are matrices formed by the left and right singular vectors of M, respectively, and Σ is a diagonal matrix composed of the singular values of M. According to the properties of singular value decomposition, all columns of U and V are mutually orthogonal. Thus, we can derive Equation (13):

(13) $$u^{T}S_{XX}^{-1/2}S_{XY}S_{YY}^{-1/2}v=u^{T}U\Sigma V^{T}v=\sigma_{uv}$$

Therefore, the maximum value of the optimization objective of Equation (12) is the maximum singular value of the matrix M. u and v are the left and right singular vectors corresponding to the largest singular values, respectively. Then, $a=S_{XX}^{-1/2}u$, $b=S_{YY}^{-1/2}v$ can be calculated.

### 2.4. Inter-Band Correlation Features Based on CCA

CCA is a multivariate statistical analysis method that aims to capture the overall correlation relationship between two sets of indicators. It does this by extracting two representative synthetic variables, U1 and V, which are linear combinations of the variables in each set. These synthetic variables are then used to reflect the overall correlation between the two sets of indicators.

The CCA algorithm involves the following steps:

1. Identify the linear combination of variables in each set that maximizes the correlation coefficient between the two sets.
2. Select an uncorrelated linear combination from the remaining options and pair it with the previously selected linear combination, selecting the one with the highest correlation coefficient.
3. Repeat steps 1 and 2 until all correlations between the two sets of indicators have been captured in the synthetic variables.

According to the CCA derivation formula in Section 2 and Section 3, when the top k singular values. singular values of the matrix M are selected, u and v are the matrices composed of the left and right singular vectors corresponding to the top k singular values, respectively. Then, $a=S_{XX}^{-1/2}u$, $b=S_{YY}^{-1/2}v$ can be calculated. At this point, Equations (4) and (5) are equivalent to a linear transformation of X and Y, so that the correlation coefficients of the two sets of random variables after the transformation are the top k singular value of the matrix M, and the linearly transformed random variables $x^{\prime }$ and $y^{\prime }$ reflect the correlation characteristics of the two original random variables.

**Figure 1:**
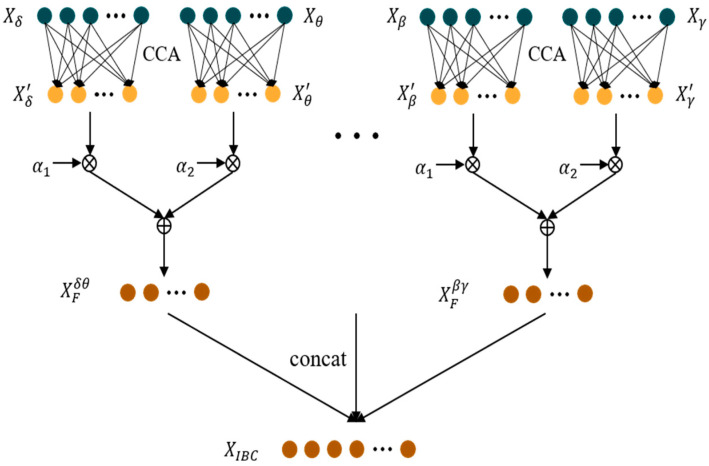
IBC feature extraction based on original DE features.

The generation of and changes in emotions usually involve multiple bands of brain activity, and there are synergies between these bands, so the inter-bands correlation (IBC) features can be extracted using CCA. The IBC calculation process is shown in Figure 1.

The DE features of five frequency bands (δ: 1–4 Hz, θ: 4–8 Hz, α: 8–13 Hz, β: 13–30 Hz, γ: 30–50 Hz) for all channels are represented as $X_{\delta }$, $X_{\theta }$, $X_{\alpha },$ $X_{\beta }$, $X_{\gamma }$, respectively. DE features of any frequency band can be treated as a set of variables, and the CCA algorithm can be used to extract the correlation between any two frequency bands of DE features. For DE features of any two frequency bands X and Y, the correlation features $X^{\prime }$ and $Y^{\prime }$ between the two frequency bands can be obtained by performing a linear transformation using CCA. As shown in Equations (14) and (15):

(14) $$X_{1}^{\prime }=W_{1}^{T}X_{1}$$

(15) $$X_{2}^{\prime }=W_{2}^{T}X_{2}$$

According to [35], we set two parameters $\alpha_{1}$ and $\alpha_{2}$, satisfying $\alpha_{1}+\alpha_{2}=1$, and perform a weighted summation of the correlation features between the two frequency bands obtained above, as shown in Equation (16):

(16) $$X_{F}=\alpha_{1}X^{\prime }+\alpha_{2}X_{2}^{\prime }$$

Thus, we obtain the IBC features $X_{F}$ between the two frequency bands. As depicted in Figure 1, for $X_{\delta }$ and $X_{\theta }$, we can obtain the correlation features $X_{\delta }^{\prime }$ and $X_{\theta }^{\prime }$ by Equations (14) and (15). Then the IBC features $X_{F}^{\delta \theta }$ between δ and θ frequency bands can be obtained by Equation (16). For the five frequency bands of the EEG, features similar to $X_{F}^{\delta \theta }$ can be obtained between any two frequency bands, so we can obtain 10 sets of features like $X_{F}^{\delta \theta }$. Finally, we concatenate 10 sets of features including $X_{F}^{\delta \theta },X_{F}^{\delta \alpha },X_{F}^{\delta \beta },X_{F}^{\delta \gamma }{,X_{F}^{\theta \alpha },X_{F}^{\theta \beta },X_{F}^{\theta \gamma },X_{F}^{\alpha \beta },X_{F}^{\alpha \gamma },X}_{F}^{\beta \gamma }$, which is the final inter-band correlation feature $X_{IBC}$, as shown in Equation (17):

(17) $$X_{IBC}=\left[X_{F}^{\delta \theta },X_{F}^{\delta \alpha },X_{F}^{\delta \beta },\ldots {,X}_{F}^{\beta \gamma }\right]$$

### 2.5. Decision-Level Fusion

Considering that some of the original information may be lost when the IBC features are obtained from the original DE features, a decision-level fusion method [15] is adopted for the original DE features and the IBC features to classify emotions. For each of these two features, a classifier is trained using a linear kernel SVM. The output of the linear kernel SVM is a vector, and each value of the vector means a probability estimate, representing the probability that the sample belongs to a certain category. The SVM-based decision fusion method can improve the accuracy and robustness of classifiers by weighting the results of multiple classifiers. Let $\omega_{k}(k=1,2,\ldots ,c)$ represent the category of emotions and $y_{DE}$ and $y_{IBC}$ represent the labels of emotions predicted by the classifier, respectively. The result of decision-level fusion is shown as follows:

(18) $$\;y_{Fusion}=\underset{w_{k}}{\arg\max}\left[p\left(w_{k}|y_{DE}\right)+p\left(w_{k}|y_{IBC}\right)\right]\;\;(k=1,\;2,\ldots ,\;c)\;$$

For each of the DE features and inter-band correlation (IBC) features, a classifier is trained using a linear kernel SVM. $p\left(w_{k}|y_{DE}\right)$ and $p\;\left(w_{k}|y_{IBC}\right)(k=1,2,\ldots ,c)$ represent the confidence of the classifiers trained using DE features and trained using IBC features to identify the k-th emotion, respectively. Then, we take a weighted summation for confidence of the two SVM classifiers outputs and take the labels corresponding to the highest sum of confidence of the two classifiers outputs as the final prediction label.

### 2.6. Cross-Validation

We use the block-wise cross-validation to evaluate the classification performance. Each block contains one video of each emotion type, and each data set has five blocks. The data of one block are used as the test set, and the data of the remaining blocks are used as the training set. This process is repeated five times to ensure that each block is used as a test set. Finally, the average classification accuracy of the block-wise cross-validation is taken as the evaluation metric.

## 3. Results 

### 3.1. Validity of IBC Features

**Figure 2:**
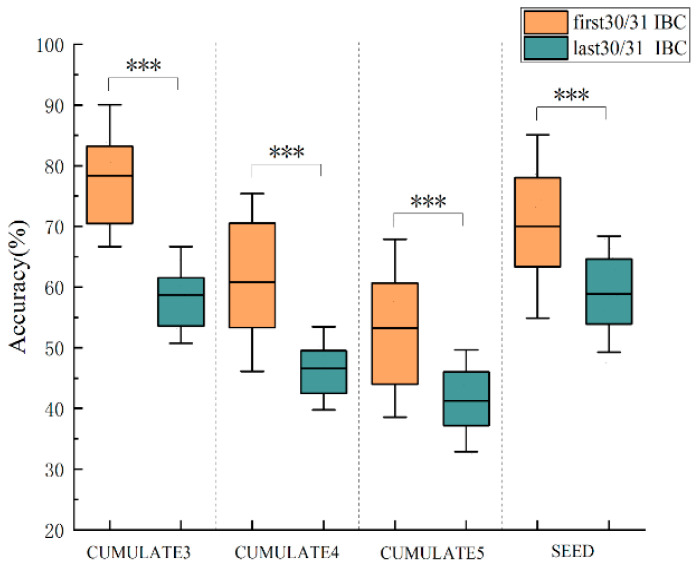
Comparison of classification accuracy between the first 30/31 IBC features and the last 30/31 IBC features on CUMULATE and SEED datasets, *** *p* < 0.001.

To ascertain the validity of the IBC features, we subjected the singular values of matrix M from the previous section to a thorough sorting in descending order. Subsequently, we selected the IBC features corresponding to the top half of these singular values and those corresponding to the bottom half, employing an SVM model for emotion classification. Specifically, for the CUMULATE dataset, we opted for the IBC features corresponding to the first 30 largest singular values and the last 30; whereas, on the SEED dataset, we selected the IBC features corresponding to the first 31 largest singular values and the last 31. In the weighted summation of correlation features across two frequency bands, we assumed equal importance for each band, hence α_1 = 0.5 and α_2 = 0.5. The outcomes of our classification are depicted in Figure 2. Specifically, CUMULATE3, CUMULATE4, and CUMULATE5 signify the three-class, four-class, and five-class datasets of CUMULATE, respectively.

Figure 2 illustrates a comparison of classification accuracy between the first 30/31 IBC features and the last 30/31 IBC features on CUMULATE and SEED datasets. A paired sample t-test was conducted to compare the classification accuracies of the first 30/31 IBC features and the last 30/31 IBC features for both datasets, respectively. The results demonstrate that the classification accuracy of the first 30/31 IBC features is significantly higher than that of the last 30/31 IBC features. For the three sub-datasets of CUMULATE, the average classification accuracy of the first 30 IBC features is 78.34%, 60.77%, and 53.23%, respectively, which is significantly higher than the corresponding average classification accuracies of the last 30 IBC features of 58.70% (*p* < 0.001), 46.61% (*p* < 0.001), and 41.27% (*p* < 0.001). On the SEED dataset, the average classification accuracy of the first 31 IBC features is 70.00%, which is significantly higher than the other half of the features corresponding to 58.83% (*p* < 0.001).

Indeed, a larger singular value corresponds to a more correlated feature between the frequency bands. Consequently, the first 30/31 IBC features are better suited to reflect the correlations between the two frequency bands compared to the last 30/31 IBC features. It can be inferred that the more correlated features between the frequency bands are more conducive to the recognition of emotions, hence the features that exhibit inter-band correlations may potentially encapsulate emotional information.

**Figure 3:**
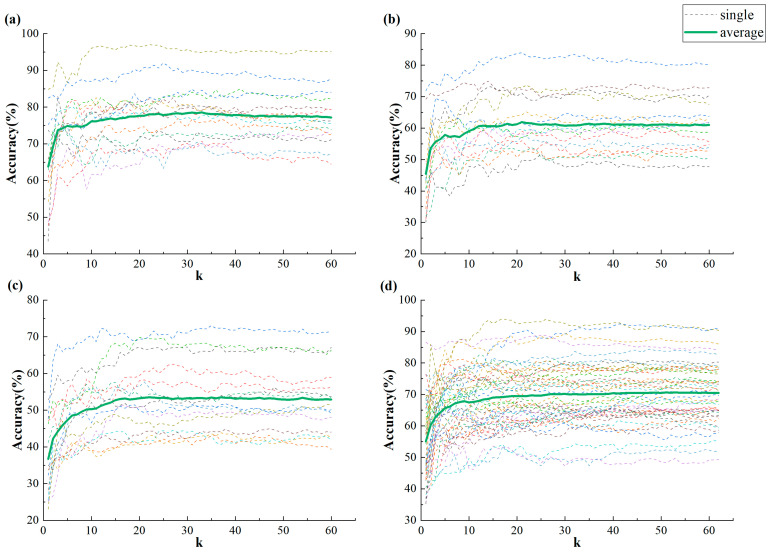
Variation curve of classification accuracy with k value on CUMULATE and SEED datasets (**a**) CUMULATE3 dataset (**b**) CUMULATE4 dataset (**c**) CUMULATE5 dataset (**d**) SEED dataset.

Furthermore, we investigate the effectiveness of training an SVM classifier with the IBC features corresponding to the top k largest singular values of M. The change curve of classification accuracy is plotted as the value of k gradually increases on both the local CUMULATE dataset (Figure 3a–c) and the public dataset SEED (Figure 3d). The dashed line and thick solid line indicate the variation in emotion recognition accuracy for each individual subject and the average classification accuracy fluctuation across all subjects, respectively.

As depicted in Figure 3, the classification accuracy demonstrates a pattern of sharp increase followed by a tendency towards flattening out across different datasets. This further underscores the notion that the correlation between frequency bands plays a crucial role in emotion recognition, with features corresponding to larger singular values being more influential. On the other hand, IBC features with smaller singular values may not be as significant in emotion recognition.

**Figure 4:**
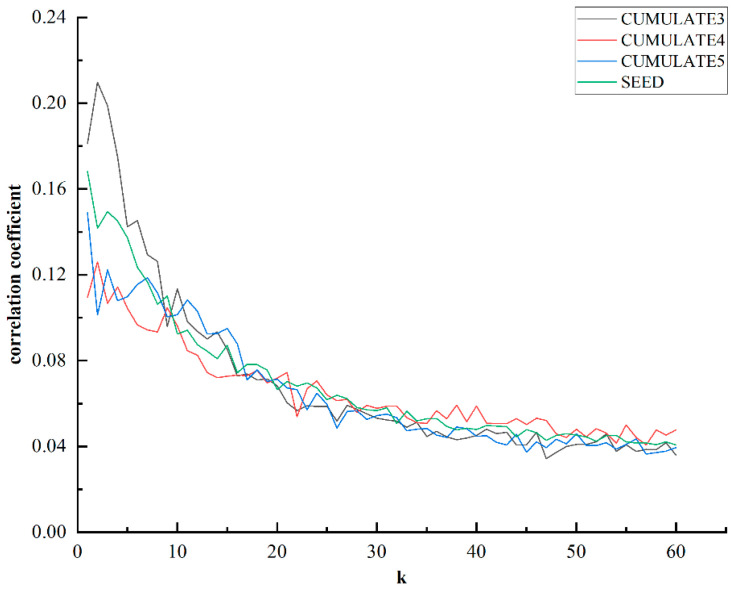
Correlation coefficient between the k-th IBC feature and emotion label.

Therefore, we conducted an analysis to investigate the correlation between the IBC features corresponding to the kth singular values and the emotion labels. The mean value of the correlation coefficient between each feature and emotion label was calculated across subjects, as shown in Figure 4.

It can be observed that the IBC features corresponding to larger singular values, which implies stronger correlations between frequency bands, exhibit a stronger correlation with emotion labels. On the other hand, the features corresponding to smaller singular values, which indicate less correlation between frequency bands, display a weaker correlation with emotion labels. This finding substantiates the notion that more correlated features between frequency bands may encompass specific emotional characteristics.

Furthermore, we designate the correlation coefficient between IBC features and emotion labels as ICC, and the correlation coefficient among different frequency bands mentioned above as DCC. To verify the validity of IBC features, we conduct an analysis of the correlation between ICC and DCC.

Table 1 and Table 2 present the correlation coefficient (CC) between ICC and DCC on the CUMULATE dataset and SEED dataset, respectively. Our analysis reveals a strong positive correlation between DCC and ICC on most subjects across both datasets. This finding indicates that as the correlation coefficient among different frequency bands increases, the corresponding IBC features exhibit a stronger association with emotion labels. We posit that emotions are intricately linked to the synergy between frequency bands, and the correlation among different frequency bands is driven by emotional factors. The IBC features corresponding to higher correlation coefficients among different frequency bands are characterized by a rich abundance of emotional information.

### 3.2. Classification Results

Based on the findings from the previous subsection, we select the IBC features corresponding to the top 30/31 singular values to train an SVM classifier. Additionally, we train another SVM classifier using the original DE features. Finally, we apply the decision-level fusion sum rule to combine the outputs of both classifiers for emotion classification.

**Figure 5:**
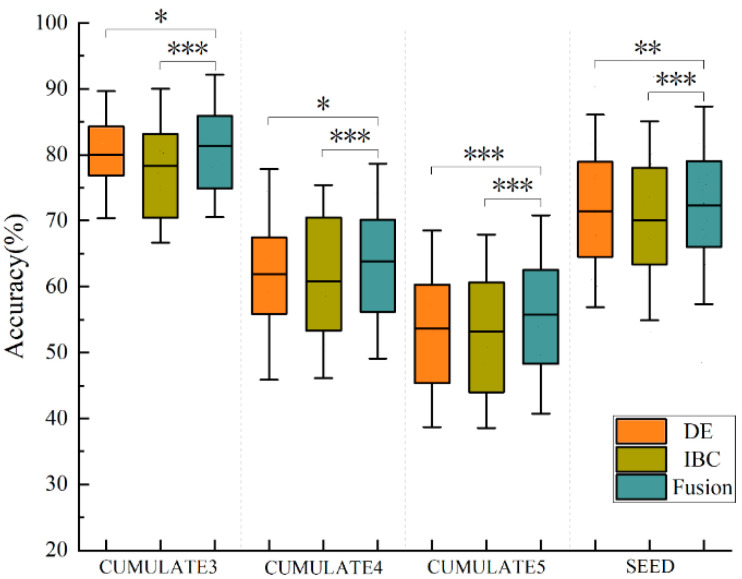
Emotion recognition accuracy using DE features, IBC features and their decision-level fusion on local CUMULATE dataset and SEED dataset, * *p* < 0.05, ** *p* < 0.01, *** *p* < 0.001.

Figure 5 displays the emotion recognition accuracy utilizing DE features, IBC features, and their decision-level fusion on both CUMULATE and SEED datasets. Notably, there is no significant difference in the accuracy of emotion recognition between the weak classifiers trained on the original DE feature and IBC feature. However, upon fusing DE and IBC features at the decision level, the classification accuracy surpasses the results obtained from individual features. For the three sub-datasets of CUMULATE, the average classification accuracies after fusion are 81.36%, 63.88%, and 55.77%, which are significantly higher than the average classification accuracies of 80.02% (*p* < 0.05), 61.86% (*p* < 0.05), and 53.61% (*p* < 0.001) using DE features alone as well as significantly higher than the average classification accuracies of mean classification accuracy of 78.34% (*p* < 0.001), 60.77% (*p* < 0.001), and 53.23% (*p* < 0.001) using IBC features alone. The same results hold true for the SEED dataset. The average classification accuracy after fusion is 72.35%, which is significantly higher than the average classification accuracy of 71.49% (*p* < 0.01) using DE features alone and higher than the average classification accuracy of 70.00% (*p* < 0.001) using IBC features alone. This outcome demonstrates that IBC features provide supplementary emotional information via decision-level fusion, thereby enhancing the accuracy of emotion recognition.

**Figure 6:**
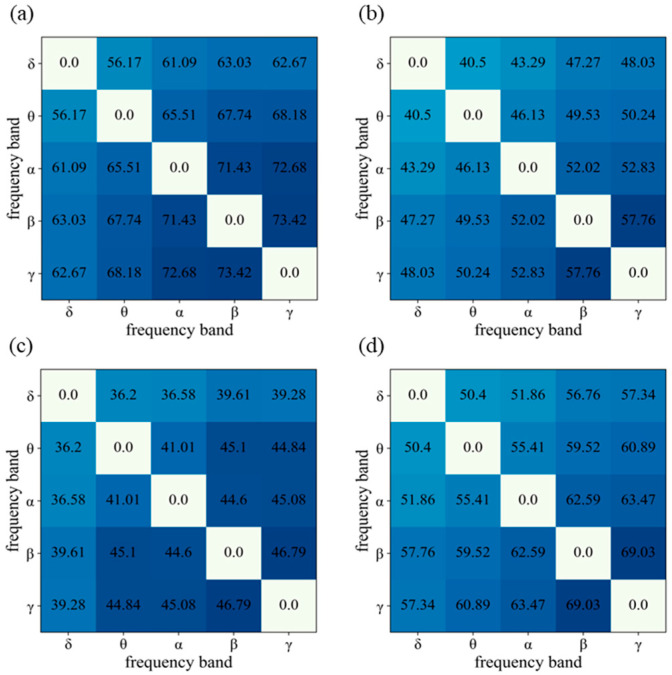
Classification accuracy of the IBC features between any two different frequency bands on local CUMULATE dataset and SEED dataset (**a**) CUMULATE3 dataset (**b**) CUMULATE4 dataset (**c**) CUMULATE5 dataset (**d**) SEED dataset.

In addition, we examine the classification accuracy of IBC features across different pairs of frequency bands. The results are presented in Figure 6, where subplots (a), (b), (c), and (d) correspond to the CUMULATE3, CUMULATE4, CUMULATE5, and SEED datasets, respectively. The diagonal elements of each subplot have been set to zero for clarity and are not considered in the analysis. Each cell in the subplot represents the classification accuracy of IBC features across different pairs of frequency bands.

The same trend is observed in all four subplots of Figure 6. The colors in the figure from light to dark means an increasing level of accuracy from low to high. The upper left part of each subplot exhibits low classification accuracy, while the lower right part demonstrates higher accuracy. This finding suggests that IBC features from higher frequency bands are more effective for emotion recognition. In particular, the correlation features between the β band and γ band yield the highest classification accuracy on each dataset, while the correlation features between the δ band and θ band provide the lowest accuracy. This result also reinforces the notion that the β and γ bands contain richer emotional information and are more effective for emotion recognition, which is consistent with the findings in [30].

## 4. Discussion

### 4.1. The Validity of IBC Features

We conducted four experiments to demonstrate the effectiveness of IBC feature extraction. Firstly, we compared the emotion classification results obtained from the IBC features corresponding to the largest half of the singular values and the IBC features corresponding to the other half of the singular values. The results showed that the emotion recognition accuracy for the first 30/31 IBC features was significantly higher than that of the last 30/31 IBC features. This indicates that most of the information related to emotions is concentrated in the first 30/31 IBC features after CCA. The emotional information contained in the last 30/31 IBC features is relatively insufficient. To further investigate this, we conducted a second experiment by selecting the IBC features corresponding to the top k largest singular values and observed the change in classification accuracy as the value of k increased gradually. The trend of change in classification accuracy was consistent across all datasets, with rapid growth at the beginning and then stabilization. This further confirms that the more correlated features between frequency bands have a greater impact on emotions, while features with weaker correlations between frequency bands contain less emotional information. In a third experiment, we directly explored the correlation between the k-th correlation feature and emotional labels. It was observed that features with a higher correlation between frequency bands had a greater correlation coefficient with emotional labels and may contain more emotional information. Finally, we performed an experiment to investigate the correlation between ICC and DCC. Our results suggest that the cross-frequency coupling features extracted using our method are potential features for emotion, and inter-bands coupling is driven by emotions.

### 4.2. Information Complementarity between IBC and DE Features

We compared the performance of emotion recognition using only DE features, IBC features, and their decision fusion on different datasets. Our results showed that the combination of DE and IBC features significantly improved the accuracy of emotion recognition compared to using either feature alone. This indicates that some information about emotions is lost when performing the IBC feature extraction via CCA based on original DE features. However, there is also information that is not included in the original DE features but is supplied by the IBC features. Therefore, the combination of these two types of features provides a complementary view of emotions, leading to better a performance in terms of emotion recognition.

### 4.3. Computational Complexity of IBC Features

Our method utilizes the SVM model and CCA for cross-frequency coupling feature extraction, avoiding surrogate testing. In terms of computational complexity, our method is more efficient than PAC coupling feature extraction, as it shortens the training time of the model. This not only improves the real-time performance of the model but also enhances its value in engineering applications. Additionally, our method contributes to higher accuracy in terms of emotion recognition.

### 4.4. Future Directions

Our proposed method has demonstrated superior results, indicating that the relevance of features across frequency bands plays a crucial role in emotion recognition. Furthermore, the underlying physiological mechanism is an important area for future research. Additionally, our feature extraction method reduces computational costs and enhances the real-time performance of the model. However, it should be noted that our approach is limited to emotion recognition research based on effective DE features. In the future, we aim to expand our algorithm ideas and apply them to other fields based on EEG signals, such as depression, anxiety [36], upper limb applications [37], and detection of epileptic seizures [38]. In doing so, we hope to contribute to a wider range of applications using EEG signals while advancing the field of emotion recognition.

## 5. Conclusions

In this paper, we present a novel approach for extracting emotional cross-band features based on differential entropy. Our method differs from traditional inter-band feature extraction methods such as phase–amplitude coupling (PAC) in that it relies on machine learning and avoids the need for surrogate testing, which can be time-consuming. This not only enhances the efficiency of our model but also improves its real-time performance. To validate the effectiveness of our proposed IBC features, we conducted experiments using two publicly available datasets: SEED and CUMULATE. The results demonstrate that combining cross-band DE features with traditional DE features outperforms methods using two single features alone, substantiating the effectiveness of our method for emotion recognition.

## Figures and Tables

**Table 1:** The correlation coefficient (CC) between ICC and DCC on CUMULATE dataset.

CUMULATE3		CUMULATE4		CUMULATE5	
Subject	CC	Subject	CC	Subject	CC
S1	0.71	S1	0.70	S1	0.76
S2	0.75	S2	0.76	S2	0.65
S3	0.74	S3	0.72	S3	0.80
S4	0.66	S4	0.57	S4	0.72
S5	0.76	S5	0.72	S5	0.81
S6	0.72	S6	0.72	S6	0.83
S7	0.76	S7	0.67	S7	0.77
S8	0.73	S8	0.66	S8	0.54
S9	0.74	S9	0.61	S9	0.59
S10	0.81	S10	0.69	S10	0.75
S11	0.72	S11	0.75	S11	0.68
S12	0.73	S12	0.62	S12	0.74
S13	0.84	S13	0.84	S13	0.83
S14	0.68	S14	0.63	S14	0.69
S15	0.85	S15	0.54	S15	0.77
AVG	0.75	AVG	0.68	AVG	0.73
STD	0.05	STD	0.07	STD	0.09

**Table 2:** The correlation coefficient (CC) between ICC and DCC on SEED dataset.

SEEDday1		SEEDday2		SEEDday3	
Subject	CC	Subject	CC	Subject	CC
S1	0.75	S1	0.66	S1	0.51
S2	0.53	S2	0.44	S2	0.75
S3	0.34	S3	0.66	S3	0.59
S4	0.78	S4	0.76	S4	0.49
S5	0.75	S5	0.82	S5	0.83
S6	0.59	S6	0.71	S6	0.67
S7	0.75	S7	0.81	S7	0.67
S8	0.58	S8	0.68	S8	0.77
S9	0.82	S9	0.81	S9	0.79
S10	0.70	S10	0.54	S10	0.78
S11	0.77	S11	0.76	S11	0.75
S12	0.81	S12	0.74	S12	0.75
S13	0.72	S13	0.76	S13	0.72
S14	0.79	S14	0.66	S14	0.67
S15	0.71	S15	0.66	S15	0.71
AVG	0.69	AVG	0.70	AVG	0.70
STD	0.13	STD	0.10	STD	0.10

## Data Availability

The local data CUMULATE that support the findings of this study are available on request from the corresponding author and the data are not publicly available due to privacy or ethical restrictions. The SEED dataset is available at https://bcmi.sjtu.edu.cn/home/seed/ (accessed on 1 March 2023).
